# Epidemiology and Resistance Phenotypes of Carbapenem-Resistant *Klebsiella pneumoniae* in Corfu General Hospital (2019–2022): A Comprehensive Time Series Analysis of Resistance Gene Dynamics

**DOI:** 10.3390/microorganisms11102537

**Published:** 2023-10-11

**Authors:** Glykeria Sorovou, Georgios Schinas, Aggeliki Pasxali, Angeliki Tzoukmani, Kyriaki Tryfinopoulou, Charalambos Gogos, George Dimopoulos, Karolina Akinosoglou

**Affiliations:** 1 Microbiology Laboratory, General Hospital of Corfu, 49100 Corfu, Greece; drkellyds@hotmail.com (G.S.); microcorfu@yahoo.gr (A.P.); 2 School of Medicine, University of Patras, 26504 Patras, Greece; georg.schinas@gmail.com (G.S.); cgogos@med.upatras.gr (C.G.); 3 Central Public Health Laboratory, National Public Health Organization, 34 Alex. Fleming Str., 16672 Vari, Greece; aggeliki_tzoukmani@hotmail.com (A.T.); k.tryfinopoulou@eody.gov.gr (K.T.); 4 3rd Department of Critical Care, Evgenidio Hospital, Medical School, National and Kapodistrian University of Athens, 11528 Athens, Greece; 5 Department of Internal Medicine and Infectious Diseases, University General Hospital of Patras, 26504 Patras, Greece

**Keywords:** *Klebsiella pneumoniae*, antimicrobial resistance, carbapenem-resistant Enterobacteriaceae, antibiotic resistance patterns, COVID-19, resistance genes, KPC, NDM, epidemiological monitoring, molecular monitoring

## Abstract

Antimicrobial resistance is a significant global health challenge, with *Klebsiella* pneumoniae being one of the most common antibiotic-resistant pathogens. This study provides an in-depth analysis of the prevalence and resistance patterns of antibiotic-resistant *Klebsiella pneumoniae* in the General Hospital of Corfu, Greece, between 2019 and 2022, with the aim of understanding the potential impact of the COVID-19 pandemic on the epidemiology of this bacterium. Utilizing a retrospective epidemiological approach, this study analyzed 212 isolates obtained from the hospital’s Microbiology Department. These isolates were subjected to genotypic and phenotypic identification, with resistance genes (bla-KPC, bla-NDM, bla-VIM, bla-OXA-48, and mcr-1) and antibiotic resistance patterns as the primary focus. The results revealed a significant shift in resistance gene prevalence, with a notable increase in bla-KPC from 16.67% in 2021 to 58.46% in 2022, and a decrease in bla-NDM from 81.48% in 2021 to 38.46% in 2022. In terms of antibiotic resistance patterns, there was a consistent increase in resistance to amikacin and a significant decrease in resistance to ceftazidime/avibactam. These findings underscore the dynamic nature of carbapenem-resistant *Klebsiella pneumoniae* (CRKP) resistance and highlight the need for ongoing surveillance and adaptive therapeutic strategies in the face of evolving resistance patterns.

## 1. Introduction

Antimicrobial resistance is a significant challenge in modern medicine, affecting sectors such as human and animal health, agriculture, and the environment [1]. The situation is exacerbated by the escalating prevalence of antibiotic resistance, which triggers extended hospital stays, amplifies medical costs, and escalates morbidity and mortality rates [2]. Factors contributing to this include the development of multidrug-resistant and extensively drug-resistant bacterial strains, the limited discovery and development of new antibiotics, and the misuse and overuse of existing antibiotics [3,4]. The global spread of antibiotic-resistant organisms is also facilitated by increased travel and trade, making the problem complex to manage [5]. In response to these pressing concerns, various strategies have been initiated, including antibiotic stewardship programs, disease prevention measures, and the novel utilization of antibiotic adjuvants or potentiators [6,7]. Collectively, these endeavors form a multifaceted defense against antibiotic resistance, yet innovative strategies have become ever-more relevant, especially following the widespread implementation of resistance genes surveillance programs [8,9].

Among the alarming manifestations of antibiotic resistance is the emergence of carbapenem-resistant *Klebsiella pneumoniae* (CRKP) strains, characterized by limited therapeutic options [10]. CRKP, characterized by its complex array of resistance markers, not only poses substantial challenges to treatment but also represents a growing global threat to public health, as evidenced by its increasing prevalence [11,12] and the high mortality rate of CRKP infections [13,14]. Diving into the molecular level, the evolution of antibiotic resistance, driven by the selective pressure exerted by antibiotics, leads to resistance through both mutations and horizontal gene transfers [15].Among the enzymes produced by *K. pneumoniae* that confer resistance against carbapenems are *Klebsiella pneumoniae* carbapenemase (KPC), New Delhi metallo-β-lactamase (NDM), and OXA-48 [16,17].

The prevailing distribution of carbapenemase-producing *K. pneumoniae* in Europe has been documented by Grundmann et al., within the framework of the European Survey on carbapenemase-producing Enterobacteriaceae (EuSCAPE), conducted between November 2013 and April 2014 across 35 European countries [18]. According to this comprehensive survey, the average occurrence of carbapenemase-producing *K. pneumoniae* or E. coli infections stood at 1.3 patients per 10,000 hospital admissions across Europe. Remarkably, in Greece, this incidence was notably higher at 5.78, positioning Greece as the second highest in prevalence, following Italy with a rate of 5.96 [18]. The molecular evolution of antibiotic resistance in Greece, possibly driven by changes in the usage of specific antibiotics or other healthcare factors, needs to be further explored. A better understanding of these potential factors could help elucidate why there has been an alarming increase in antibiotic resistance, particularly carbapenem resistance, in this region.

The aim of this study is to assess the prevalence of CRKP in the General Hospital of Corfu within the 2019–2022 timeframe, in order to understand the influence that the COVID-19 pandemic may have had on the epidemiology of this bacterium, by mapping the temporal trends of resistance through a comprehensive time series analysis. Insights from this study may reveal important connections between resistance mechanisms at genetic and phenotypic levels that could potentially enhance our understanding of the impact of CRKP antibiotic resistance in clinical settings and, most importantly, serve as the foundation for devising infection control measures and implementing prudent antibiotic usage practices.

## 2. Materials and Methods

### 2.1. Study Design and Data Collection

This retrospective epidemiological study was conducted at the Microbiology Department of General Hospital of Corfu, Greece, focusing on all carbapenem-resistant *Klebsiella pneumoniae* strains isolated from January 2019 to December 2022. The General Hospital of Corfu is a secondary care hospital with a bed capacity of 280, providing medical, surgical, and specialized care, including intensive care units, in northwestern Greece. Carbapenem resistant *Klebsiella pneumoniae* were sought, and records of relevant cases were collected, including patient demographics, clinical characteristics, antibiotic resistance profiles, resistance gene expressions, and outcomes. The study was performed according to the principles of the Declaration of Helsinki, ensuring good clinical research practice and adherence to the General Data Protection Regulation, which was approved by the local institutional review board and respective ethics research committee.

### 2.2. Genotypic and Phenotypic Identification

Identification of the strains and minimal inhibitory concentration (MIC) determination of various antibiotics, including ceftazidime/avibactam, were performed using VITEK 2, according to EUCAST recommendations. Colistin and tigecycline MICs were additionally evaluated using MBD (MERLIN) and E-test, respectively. Multiplex PCR was performed for each strain to detect the presence of bla-genes (VIM, KPC, NDM, OXA-48) and mcr-1 genes.

### 2.3. Statistical Analysis

A comprehensive descriptive analysis was performed to outline the population’s characteristics as well as the variations in antimicrobial resistance, resistance gene expression, and antimicrobial consumption over time. Trend analysis was conducted using the Cochran–Armitage Trend Test to elucidate underlying trends in antibiotic resistance and resistance genes patterns over the collection years, at annual intervals. This statistical method was applied to evaluate the significance of trends, providing definitive insights into the temporal evolution of resistance characteristics in the *Klebsiella* isolates. Moreover, a correlation analysis was performed to explore the relationships between antibiotic resistance and resistance gene expression using mean values across years. Line graphs were constructed to represent variations in resistance, gene expression, and antimicrobial consumption over time. Six-month intervals served as the time unit for these visualizations. Statistical tests tailored to the data distribution were employed. The statistical analysis was conducted utilizing IBM SPSS Statistics, Version 26 (IBM Corp., Armonk, NY, USA) and STATA Version 16 (StataCorp LLC, College Station, TX, USA). A significance level of α < 0.05 was adopted for all statistical tests.

## 3. Results

### 3.1. Population Characteristics 

The study population comprised 212 isolates, with a significant majority obtained from the medical department (76%), followed by the ICU (12.2%), and the surgical department (11.8%). The median patient age was 81 years, and 47.2% of the patients were male. The most common sample type was urine (72.6%), and 25.9% had a positive blood culture result. Comorbidities were present in various proportions, with diabetes mellitus (10.4%) and hypertension (8%) being the most prevalent comorbidities. A recorded total of 28 patients (13.2%) succumbed to their illness (Table 1).

### 3.2. Prevalence of Resistance Genes (2019–2022)

The prevalence of different resistance genes, including bla-KPC, bla-NDM, bla-VIM, bla-OXA-48, and mcr-1, was evaluated across the collection years. Only two of our patients expressed a double mechanism of resistance. A significant increase in bla-KPC and decline in bla-NDM were noted, with variations in other genes (Table 2).

### 3.3. Antibiotic Resistance Patterns (2019–2022)

**Figure 1:**
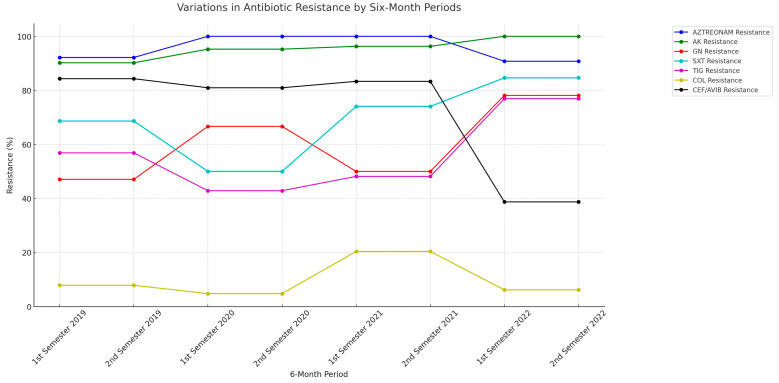
Line Graph Illustrating Variations in Antibiotic Resistance (2019–2022). Note: Temporal Variations in Antibiotic Resistance by Six-Month Periods from January 2019 to December 2022. The line graph illustrates the percentage of antibiotic resistance for selected agents stratified by six-month intervals. Each line represents a distinct antibiotic agent, and the color codes for the lines are indicated in the legend. The *X*-axis denotes the six-month periods, while the *Y*-axis represents the percentage of resistance. Abbreviations: amikacin (AK), gentamycin (GN), co-trimoxazole (SXT), tigecycline (TIG), colistin (COL), ceftazidime/avibactam (CEF/AVIB).

This study elucidated the patterns of resistance to various antibiotics, including aztreonam, amikacin (AK), gentamycin (GN), co-trimoxazole (SXT), tigecycline (TIG), colistin (COL), and ceftazidime/avibactam (CEF/AVIB). Notable trends included a consistent increase in AK resistance, a significant drop in CEF/AVIB resistance, and fluctuations in other antibiotics (Table 3, Figure 1). Trend analysis, using the Cochran–Armitage Trend Test, revealed statistically significant trends in most antibiotics, with increasing resistance in AZTREONAM, AK, GN, SXT, and TIG, fluctuating resistance in COL, and decreasing resistance in CEF/AVIB (Supplementary Table S1).

### 3.4. Resistance Genes Patterns (2019–2022)

**Figure 2:**
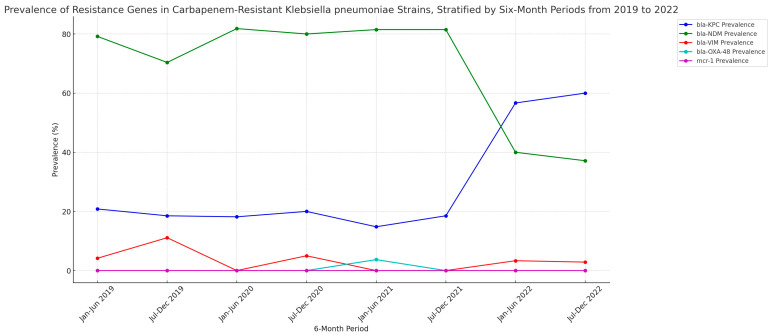
Line graph illustrating variations in resistance gene patterns (2019–2022). Note: Prevalence of Resistance Genes in Carbapenem-Resistant Klebsiella pneumoniae Strains from 2019 to 2022. The line graph represents the prevalence (%) of five key resistance genes (bla-KPC, bla-NDM, bla-VIM, bla-OXA-48, mcr-1) stratified by six-month periods. Data pertaining to specific percentages across six-month intervals can be found in Supplementary Table S3.

The temporal evolution of resistance genes is depicted in Figure 2, highlighting a significant increase in bla-KPC, a decline in bla-NDM, and fluctuations in other genes. The Cochran–Armitage Trend Test also identified trends in resistance genes, with an increasing trend for bla-KPC, a decreasing trend for bla-NDM, fluctuating trends for bla-VIM and bla-OXA-48, and a constant presence of mcr-1 (Supplementary Table S2).

### 3.5. Antibiotic Consumption Patterns (2019–2022)

**Figure 3:**
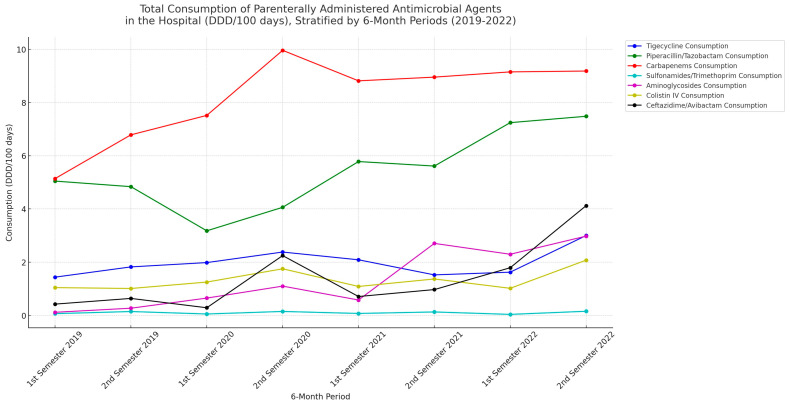
Trends in Antimicrobial Consumption at General Hospital of Corfu (2019–2022). Note: The line graph represents the total consumption of parenterally administered antimicrobial agents, measured in Defined Daily Doses (DDD) per 100 days of hospitalization, for each six-month period from the first semester of 2019 to the first semester of 2022. Each line corresponds to a specific antimicrobial agent, depicted in unique colors for better visual distinction.

A marked increase in antibiotic consumption of all regimens except for SXT was observed between 2019 and 2022. Pip/tazo and carbapenems were dominating in use (Table 4, Figure 3). 

## 4. Discussion

In Greece, the epidemiology of CRKP has exhibited marked variations across regions and hospitals. Before the year 2001, the Greek Antimicrobial Resistance Surveillance System recorded a prevalence of carbapenem resistance at less than 1%. The resistance among carbapenem-resistant Enterobacteriaceae (CRE) was primarily linked to the presence of VIM-1–type metallo-β-lactamases (MBL). The resistance of *K. pneumoniae* to carbapenem antibiotics, due to the presence of VIM enzymes, was followed by the development of *K. pneumoniae* carbapenemase (KPC) production, both of which have become widespread [19,20]. The landscape changed dramatically by 2008, when the prevalence of CRE in Greece surged to 30% in hospital wards and 60% in intensive care units (ICUs) [21]. As of 2014, the European Centre for Disease Prevention and Control (ECDPC), through its European Antimicrobial Resistance Surveillance Network (EARS-Net), reported that at least 62.3% of all *K. pneumoniae* isolates collected from invasive infections were resistant to carbapenems.

The emergence of NDM-producing strains was first documented in 2011 at the University Hospital of Ioannina in Epirus, Central Greece [22]. Since then, sporadic incidents have been noted in hospitals located in Athens (Attica), Patras (western Greece), and Larissa (central Greece). These incidents are all linked to the multilocus sequence type (ST) 11 [23,24,25]. The initial outbreak, involving OXA-48-like carbapenemase, was identified in 2012 [26], and apart from isolated cases [27,28], no significant widespread outbreaks have been registered in Greece. According to the most recent yearly surveillance report released by the European Centre for Disease Prevention and Control (ECDC) [29], Greece had the highest proportion of carbapenem-resistant isolates among invasive *K. pneumoniae* cases in Europe. Alarmingly, there has been an elevating trend, with the percentage increasing from 64.7% in 2017 to 73.7% in 2021.

Within Grundmann’s survey [18], out of the 86 *K. pneumoniae* isolates displaying non-susceptibility to carbapenems in Greece, a significant portion (65%) were identified as KPC-positive, making it the most prevalent type. Following KPC, the distribution included NDM (14%), VIM (11%), and OXA-48 (2%) positive isolates [18]. In a more recent multicenter study conducted by Galani et al., involving 394 *K. pneumoniae* isolates resistant to carbapenems from 15 hospitals in Greece, the distribution showcased 66.5% as KPC-producers, 13.7% as NDM-producers, 8.6% as VIM-producers, 5.6% as dual KPC and VIM-producers, and 3.6% as OXA-48-producers [30]. Similarly, in another report within the ICUs of a tertiary hospital in Thessaloniki, Greece between 2016 and 2019, KPC was also the predominant carbapenemase; NDM were also present, and only a few double-carbapenemase producers were isolated [31]. The previous reports also showed that despite the national predominance of KPC-producing *K. Pneumoniae*, metallo-β-lactamases are the main genotype in Corfu hospital [30]. More specifically, the presence of KPC- versus NDM-producing isolates during the period 2014–2016 were 14.3 vs. 81.0%, respectively [30]. Contrary to rest of Greek Hospitals, this comes in line with neighboring University Hospital of Ioannina underlining the risk of wide antimicrobial spread through patient transfer within the community or health care facilities. Of note, the scenario in Greece stands in stark contrast to that of various other European nations like Spain, France, Germany, Turkey, Romania, and Belgium. In these countries, OXA-48, primarily associated with healthcare sources related to community-onset cases, ranks as the most commonly observed type of carbapenemase [18,32,33,34,35].

Our study uncovered marked variations in the prevalence of resistance genes over the four-year period, as we observed a notable epidemiological shift in the post-2021 period, characterized by a transition from NDM to KPC dominance. According to our findings, the occurrence of bla-KPC increased from a prevalence of 14.81% in the first half of 2021 to an astounding 60% in the last half of 2022. On the other hand, the prevalence of bla-NDM decreased from 81.48% in the first half of 2021 to just 37.14% in the last six months of 2022. Furthermore, bla-VIM prevalence remained low throughout, while bla-OXA-48 was detected in just 3.7% of cases in the first half of 2021, and mcr-1 was not detected at all in the given time frame. These shifts in resistance gene prevalence may be indicative of underlying shifts in resistance mechanisms, potentially shaped by factors such as patient transfer between neighboring hospitals due to limited bed capacity during the COVID-19 pandemic. In the recent past, the epidemiological alignment of the General Hospital of Corfu with the neighboring University Hospital of Ioannina, where the emergence of NDM-producing strains was first reported, is indicative of such a dispersion pattern. Especially in the case of ICU beds, patient translocation would exceed neighboring health care facilities, and involve the critically ill all across the country, hence explains the KPC increase, in the context of a wider epidemiological landscape of CRKP in Greece. Even though no other policy change occurred within this hospital during this period and this mechanism is plausible, further data on patient handling within the healthcare system during the pandemic are necessary for solid conclusions to be drawn. 

Managing infections caused by carbapenem-non-susceptible *K. pneumoniae* poses a significant clinical hurdle due to the lack of viable alternative medications. These options are often constrained by limited bactericidal effectiveness and/or pronounced toxicity. Among the remaining choices at the disposal of healthcare professionals to combat these challenging infections are aminoglycosides, tigecycline, colistin and fosfomycin [36], as well as the recently approved ceftazidime/avibactam, imipenem/cilastatin/relebactam and meropenem/vaborbactam combinations [37]. These antimicrobial agents, both alone and in various combinations with or without an older regimen, are integrated into therapeutic strategies determined by factors such as the microbial minimum inhibitory concentration (MIC), the location and seriousness of the infection, and the specific carbapenemase-encoding genes involved [38]. 

The antibiotic resistance patterns we observed in this study revealed complex dynamics, with a consistent increase in resistance to certain antibiotics, such as AK, and a significant drop in resistance to CEF/AVIB, in line with the drop in metallo-β-lactamase producers. As expected, a time-lag effect on resistance patterns is observed in relation to antibiotic consumption. For example, an increase in the consumption of specific antimicrobials may not immediately result in increased resistance but may manifest in subsequent six-month periods. This delayed effect could be attributed to various factors including microbial adaptation time and persistence of the antibiotic within the healthcare environment. In our work, an uptick in the consumption of carbapenems in the second semester of 2021 is followed by a significant increase in resistance (measured by bla-KPC prevalence) in the first semester of 2022. This suggests that increased antibiotic usage may precipitate resistance, albeit with a temporal lag. This six-month lag could indicate a delayed response in gene expression to changes in antimicrobial consumption. The onset of the COVID-19 pandemic in early 2020 led to a general increase in antimicrobial consumption, presumably for empirical treatments. It is crucial, though, to recognize that these observations are correlative and not necessarily causative. However, they do highlight the importance of continuous monitoring and judicious antimicrobial prescribing to combat rising resistance. The data support the concept that increased antimicrobial prescription often correlates with rising resistance, thereby affirming the importance of antimicrobial stewardship initiatives.

The use of ceftazidime/avibactam exhibited effectiveness against 99.7% of KPC/OXA-48 *K. pneumoniae* isolates. Among *K. pneumoniae* in Greek hospitals, the primary factor contributing to carbapenem resistance is the production of KPC, and avibactam seems to effectively inhibit this carbapenemase. As a result, the ceftazidime/avibactam combination presents a valuable therapeutic choice for infections caused by KPC-producing *K. pneumoniae* isolates, even those resistant to most other antimicrobial drugs [38,39]. However, proper understanding of the underlying resistance mechanism is imperative before its application [38]. In our study, the incorporation of ceftazidime/avibactam into the therapeutic regimen for CRKP infections in Corfu, conducted in compliance with rigorous antimicrobial stewardship protocols, did not lead to an escalation in NDM resistance patterns, contrary to the assumptions posited by earlier studies [40,41].

As reported in the nationwide surveillance conducted by Maltezou et al., it was noted that the resistance rates for colistin, gentamicin, and tigecycline among carbapenem-resistant *K. pneumoniae* were 23.0%, 19.7%, and 22.4%, respectively [42]. A later nationwide study by Galani et al. described a rate of non-susceptibility to colistin of 40.4% [30]. Within our collection of isolates, the rate of non-susceptibility to colistin ranged from 4.76 to 20.37%, according to the year assessed. This is likely linked to chromosomal mutations, as no instances of mcr-1-positive isolates were detected among the strains exhibiting non-susceptibility, throughout the years. Comparatively, the average non-susceptibility rate for colistin is lower than the 28.3% reported by the EuSCAPE project, which encompassed 36 participating countries [18]. Our results agree with similar trends of low colistin resistance but high and rising tigecycline resistance from the Greek Electronic System for the Surveillance of Antimicrobial Resistance—WHONET-Greece—between January 2018 and March 2021 [43]. 

We found a very high and rising resistance to amikacin and gentamycin, respectively, among CRKP isolates. Within Greece, the prevailing resistance to aminoglycosides in clinical isolates of CRKP is primarily attributed to the presence of aminoglycoside-modifying enzymes (AMEs), accounting for 85.3% of the isolates. In contrast, the presence of ribosomal methyltransferases (RMTs) has been detected in 7.7% of the isolates [44]. This scenario highlights the predominant role of AMEs in this context. Notably, a significant range of diversity has been observed in AME patterns. In total, 23 distinct AME patterns have been identified, each associated with varying levels of aminoglycoside resistance. These patterns encompassed up to five genes per isolate, underscoring the complexity of aminoglycoside resistance mechanisms [44].

This study, while providing valuable insights into the shifting epidemiology of CRKP in Corfu General Hospital, presents several limitations that should be acknowledged. Firstly, the study is confined to a single hospital, limiting the generalizability of the findings to broader regional or national contexts. The transition observed in resistance genes from NDM to KPC dominance post-2022 is noteworthy but may not represent a universal trend. Secondly, the analysis is restricted to the period from 2019 to 2022, which may not capture the complete evolutionary trajectory of CRKP, particularly in the context of the COVID-19 pandemic. Thirdly, the study relies on existing records and patient data, possibly introducing biases in sample selection, data availability, and information accuracy. The lack of patient-level data restricts us from making inferences about individual risk factors for developing resistance. As a result, while the correlations observed are suggestive, they are not confirmatory. They point toward an avenue for future research, where controlled prospective studies could provide more definitive answers. Finally, the study’s focus on specific resistance genes and antibiotics may overlook other relevant resistance mechanisms or therapeutic considerations. Notably, during the undertaking of this study, imipenem/cilastatin/relebactam and meropenem/vaborbactam had not been launched in Greece, while fosfomycin was not available in this hospital, hence not included in this analysis. Moreover, our analysis did not allow for clonal identification. Such further detection would be useful as to the understanding of the spread and presence of specific resistance mechanisms, as previously shown with ST11 blaNDM [22,23,25,30]. Despite these limitations, the study contributes significantly to the understanding of CRKP’s temporal trends and resistance gene dynamics, with potential implications for antibiotic stewardship and infection control in the post-COVID era.

The results of this study provide a comprehensive overview of the temporal trends in antibiotic-resistant *Klebsiella pneumoniae* within the General Hospital of Corfu. These trends underscore the dynamic nature of CRKP resistance in the clinical setting of Corfu General Hospital and may indicate the necessity for re-evaluating therapeutic approaches, particularly in light of the profound changes observed post-2022. Real-time study of this evolutionary process and the application of whole-genome sequencing (WGS)-based approaches offer promising pathways for tracking and identifying the emergence and proliferation of genes associated with antibiotic resistance [45]. At the same time, our study highlights the potential that seemingly irrelevant exogenous factors like COVID-19 may have a butterfly effect on whole different areas of clinical practice in an otherwise “closed” healthcare facility, requiring constant resistance genotype surveillance, antimicrobial stewardship, and adaptive therapeutic strategies. Future work should aim to incorporate more variables, such as patient-level factors and clinical outcomes, to build a comprehensive model that can better predict resistance patterns based on antibiotic consumption and other influencing elements. Furthermore, conducting a multivariate analysis of potential risk factors would provide a more robust understanding of the multiple factors that contribute to antibiotic resistance.

## Figures and Tables

**Table 1:** Demographic and Clinical Characteristics of the Study Population (n = 212).

Department, n (%)	
Medical	161 (76)
ICU	26 (12.2)
Surgical	25 (11.8)
Male sex, n (%)	100 (47.2)
Age, median (IQR)	81 (72–88)
Type of samples, n (%)	
BAL	11 (5.2)
CVC	5 (2.4)
Rectal swab	12 (5.6)
Sputum	7 (3.3)
Trauma	8 (3.7)
Urine	154 (72.6)
Other	15 (7.2)
Positive BCx, n (%)	55 (25.9)
Comorbidities, n (%)	
CAD	14 (6.6)
CKD	6 (2.8)
CVA	10 (4.7)
DM	22 (10.4)
Hypertension	17 (8)
HF	5 (2.3)
Cancer	9 (4.2)
Recent Hospitalization, n (%)	48 (22.6)
Death, n (%)	28 (13.2)
Year Cx obtained	
2019	51 (24)
2020	42 (19.8)
2021	54 (25.6)
2022	65 (30.6)

Abbreviations: ICU; Intensive Care Unit, IQR; Interquartile Range, BAL; Bronchoalveolar Lavage, CVC; Central Venous Catheter, BCx; Blood Culture, CAD; Coronary Artery Disease, CKD; Chronic Kidney Disease, CVA; Cerebrovascular Accident, DM; Diabetes Mellitus, HF; Heart Failure.

**Table 2:** Prevalence of Resistance Genes from 2019 to 2022.

Collection Year	bla-KPC	bla-NDM	bla-VIM	bla-OXA-48	mcr-1
2019	10 (19.61%)	38 (74.51%)	4 (7.84%)	0 (0.0%)	0 (0.0%)
2020	8 (19.05%)	34 (80.95%)	1 (2.38%)	0 (0.0%)	0 (0.0%)
2021	9 (16.67%)	44 (81.48%)	0 (0.0%)	1 (1.85%)	0 (0.0%)
2022	38 (58.46%)	25 (38.46%)	2 (3.08%)	0 (0.0%)	0 (0.0%)

Note: The table presents the frequencies and the percentage distribution of different resistance genes (bla-KPC, bla-NDM, bla-VIM, bla-OXA-48, mcr-1) across the years 2019 to 2022. The percentages indicate the presence of these genes within the analyzed samples.

**Table 3:** Antibiotic Resistance Patterns.

Antibiotic	Year	bla-KPC (%)	bla-NDM (%)	bla-OXA-48 (%)
AK Resistance	2019 (n = 51)	17.6	66.7	0.0
	2020 (n = 42)	16.7	78.6	0.0
	2021 (n = 54)	16.7	77.8	1.9
	2022 (n = 65)	58.5	38.5	0.0
Aztreonam Resistance	2019 (n = 51)	19.6	66.7	0.0
	2020 (n = 42)	19.0	81.0	0.0
	2021 (n = 54)	16.7	81.5	1.9
	2022 (n = 65)	58.5	29.2	0.0
CEF/AVIB Resistance	2019 (n = 51)	3.9	74.5	0.0
	2020 (n = 42)	2.4	78.6	0.0
	2021 (n = 54)	0.0	81.5	1.9
	2022 (n = 65)	0.0	33.8	0.0
COL Resistance	2019 (n = 51)	2.0	5.9	0.0
	2020 (n = 42)	0.0	4.8	0.0
	2021 (n = 54)	7.4	13.0	0.0
	2022 (n = 65)	3.1	3.1	0.0
GN Resistance	2019 (n = 51)	5.9	35.3	0.0
	2020 (n = 42)	2.4	64.3	0.0
	2021 (n = 54)	11.1	38.9	0.0
	2022 (n = 65)	49.2	24.6	0.0
SXT Resistance	2019 (n = 51)	9.8	52.9	0.0
	2020 (n = 42)	16.7	33.3	0.0
	2021 (n = 54)	14.8	59.3	0.0
	2022 (n = 65)	52.3	30.8	0.0
TIG Resistance	2019 (n = 51)	9.8	41.2	0.0
	2020 (n = 42)	9.5	33.3	0.0
	2021 (n = 54)	11.1	37.0	0.0
	2022 (n = 65)	43.1	32.3	0.0

Amikacin (AK), gentamycin (GN), co-trimoxazole (SXT), tigecycline (TIG), colistin (COL), ceftazidime/avibactam (CEF/AVIB).

**Table 4:** Total consumption of parenterally administered antimicrobial agents in the hospital (DDD/100 days of hospitalization), stratified by 6-month periods (2019–2022).

Antimicrobial Agents	1st Semester 2019	2nd Semester 2019	1st Semester 2020	2nd Semester 2020	1st Semester 2021	2nd Semester 2021	1st Semester 2022	2nd Semester 2022
Τigecycline	1.438	1.823	1.985	2.381	2.090	1.523	1.625	3.001
Piperacillin/Tazobactam	5.047	4.838	3.179	4.062	5.781	5.612	7.243	7.482
Monobactams/Aztreonam	0	0	0	0	0	0	0	0
Carbapenems (Meropenem, Ertapenem, Doripenem, Imipenem)	5.141	6.785	7.515	9.960	8.814	8.953	9.150	9.182
Sulfonamides/Trimethoprim	0.067	0.146	0.054	0.149	0.074	0.130	0.039	0.156
Macrolides (Erythromycin, Clarithromycin, Azithromycin)	0.914	0.947	1.582	1.873	0.565	0.371	0.655	0.274
Clindamycin	3.031	3.250	2.655	3.422	3.558	3.801	3.453	4.681
Aminoglycosides (Streptomycin, Amikacin, Gentamicin, Tobramycin)	0.118	0.271	0.651	1.101	0.579	2.706	2.298	2.979
Quinolones (Ciprofloxacin, Levofloxacin, Ofloxacin, Moxifloxacin)	7.410	4.978	7.731	7.141	6.512	11.542	10.474	3.872
Glycopeptides (Vancomycin, Teicoplanin, Dalbavancin)	2.891	3.988	4.736	3.374	4.007	4.297	4.816	4.975
Colistin IV	1.045	1.012	1.253	1.752	1.088	1.370	1.019	2.076
Linezolid-Tedizolid	1.132	1.083	1.288	1.194	1.422	1.255	1.327	1.189
Ceftolozane/Tazobactam	0.620	0.446	0.571	1.854	0	0	0.554	1.156
Ceftazidime/Avibactam	0.425	0.639	0.288	2.248	0.710	0.971	1.792	4.123

## Data Availability

Data are available upon reasonable request to the corresponding authors.

## Supplementary Materials

The following supporting information can be downloaded at: https://www.mdpi.com/article/10.3390/microorganisms11102537/s1, Table S1: Trends in Antibiotic Resistance Patterns for *Klebsiella* Isolates (2019–2022); Table S2: Trends in Resistance Genes Patterns for *Klebsiella* Isolates (2019–2022). Table S3: Prevalence of Resistance Genes in Carbapenem-Resistant Klebsiella pneumoniae Strains, Stratified by Six-Month Periods from 2019 to 2022.
