# Supplementary material for: Epidemiology and Resistance Phenotypes of Carbapenem-Resistant Klebsiella pneumoniae in Corfu General Hospital (2019–2022): A Comprehensive Time Series Analysis of Resistance Gene Dynamics
Source: Microorganisms. 2023 Oct 11;11(10):2537. doi: 10.3390/microorganisms11102537 (PMC10608955; doi:10.3390/microorganisms11102537)
Supplement: Supplementary file 1 [file microorganisms-11-02537-s001.zip › microorganisms-2614990-supplementary.pdf]

Supplementary Table S1: Trends in Antibiotic Resistance Patterns for Klebsiella Isolates (2019-2022)		
Antibiotic Resistance	Trend Direction	p-value
AZTREONAM RESISTANCE	Increasing	0.03196
AK RESISTANCE	Increasing	0.07770
GN RESISTANCE	Increasing	0.00728
SXT RESISTANCE	Increasing	0.00158
TIG RESISTANCE	Increasing	0.00131
COL RESISTANCE	Fluctuating	0.02749
CEF/AVIB RESISTANCE	Decreasing	1.99×10^{-8}
Note: The table represents the Cochran-Armitage Trend Test results for antibiotic resistance patterns in Klebsiella isolates over the years 2019 to 2022. The p-value tests the hypothesis that there is no trend, with values below 0.05 indicating statistical significance. Interpretation of the trend direction should be aligned with observed frequencies.		

Supplementary Table S2: Trends in Resistance Genes Patterns for Klebsiella Isolates (2019-2022)		
Resistance Genes	Trend Direction	p-value
bla-KPC	Increasing	1.80×10^{-7}
bla-NDM	Decreasing	2.19×10^{-7}
bla-VIM	Fluctuating	0.1537
bla-OXA-48	Fluctuating	0.4010
mcr-1	Constant	1.0000
Note: The table represents the Cochran-Armitage Trend Test results for resistance genes patterns in Klebsiella isolates over the years 2019 to 2022. The p-value tests the hypothesis that there is no trend, with values below 0.05 indicating statistical significance. Interpretation of the trend direction should be aligned with observed frequencies.		

Supplementary Table S3: Prevalence of Resistance Genes in Carbapenem-Resistant Klebsiella pneumoniae Strains, Stratified by Six-Month Periods from 2019 to 2022					
6-Month Period	bla-KPC	bla-NDM	bla-VIM	bla-OXA-48	mcr-1
Jan-Jun 2019	5 (20.83%)	19 (79.17%)	1 (4.17%)	0 (0.00%)	0 (0.00%)
Jul-Dec 2019	5 (18.52%)	19 (70.37%)	3 (11.11%)	0 (0.00%)	0 (0.00%)
Jan-Jun 2020	4 (18.18%)	18 (81.82%)	0 (0.00%)	0 (0.00%)	0 (0.00%)
Jul-Dec 2020	4 (20.00%)	16 (80.00%)	1 (5.00%)	0 (0.00%)	0 (0.00%)
Jan-Jun 2021	4 (14.81%)	22 (81.48%)	0 (0.00%)	1 (3.70%)	0 (0.00%)
Jul-Dec 2021	5 (18.52%)	22 (81.48%)	0 (0.00%)	0 (0.00%)	0 (0.00%)
Jan-Jun 2022	17 (56.67%)	12 (40.00%)	1 (3.33%)	0 (0.00%)	0 (0.00%)
Jul-Dec 2022	21 (60.00%)	13 (37.14%)	1 (2.86%)	0 (0.00%)	0 (0.00%)
Note: The table delineates the prevalence of resistance genes (bla-KPC, bla-NDM, bla-VIM, bla-OXA-48, mcr-1) in K. Pneumoniae multidrug resistant isolates for each six-month interval from January 2019 to December 2022.					
